# Inducible nitric oxide synthase response and associated cytokine gene expression in the spleen of mice infected with *Clonorchis sinensis*

**DOI:** 10.1007/s00436-015-4347-9

**Published:** 2015-02-17

**Authors:** Ji-Qing Shen, Qing-Li Yang, Yan Xue, Xiao-Bing Cheng, Zhi-Hua Jiang, Yi-Chao Yang, Ying-Dan Chen, Xiao-Nong Zhou

**Affiliations:** 1 National Institute of Parasitic Disease, Chinese Center for Disease Control and Prevention; Key Laboratory of Parasite and Vector Biology, MOH, WHO Collaborating Centre for Malaria, Schistosomiasis and Filariasis, Shanghai, 200025 People’s Republic of China; 2 Department of Parasitology, Guangxi Medical University, Nanning, 530021 People’s Republic of China; 3 Guangxi Zhuang Autonomous Region Center for Disease Prevention and Control, Guangxi Key Laboratory for the Prevention and Control of Viral Hepatitis, Nanning, 530028 People’s Republic of China

**Keywords:** *Clonorchis sinensis*, Spleen, Inducible nitric oxide synthase, Th1

## Abstract

*Clonorchis sinensis* is a food-borne parasite that induces a permanent increase of nitrosation in the body upon infection. The spleen is an important secondary lymphoid organ for the regulation of immune responses locally and in the whole body. However, the functions and mechanisms of the spleen in nitric oxide (NO) responses after *C. sinensis* infection remain unknown. In this study, BALB/c mice were infected with 20, 40, and 80 *C. sinensis* metacercariae to simulate mild, moderate, and severe infections, respectively. We examined the expression of inducible nitric oxide synthase (iNOS) in the spleen and the relevant cytokine transcription in splenocytes from the mice infected with different amounts of metacercariae. The iNOS of the mice infected with 80 metacercariae was expressed in the spleen as early as 10 days post-infection (dpi) and gradually increased until 90 dpi. The iNOS expression in the mice infected with 40 metacercariae was detected only at 45 and 90 dpi, but not in the mice infected with 20 metacercariae. The level of interferon (IFN)-γ messenger RNA (mRNA) transcription in splenocytes significantly increased at 10 and 20 dpi (*P* < 0.05) in response to mild/moderate infection but gradually decreased to normal levels after 45 dpi. The level of IL-12p35 mRNA transcription did not change at 10 and 20 dpi but significantly decreased after 45 dpi under moderate/severe infection (*P* < 0.05/0.01/0.001). The level of IL-18 mRNA transcription significantly increased at 10 dpi (*P* < 0.05/0.01) but significantly decreased after 20 dpi (*P* < 0.05/0.01/0.001). These results suggest that spleen is an important organ for iNOS/NO responses, which correspond to the severity of *C. sinensis* infection, but cannot be attributed to the expression of the Th1 cytokines.

## Introduction

*Clonorchis sinensis* is the causative agent of clonorchiasis, a hepatobiliary disease that epidemiologically occurred in Southern China, Korea, Taiwan, and Vietnam (Lun et al. 2005; Rim 2005; Qian et al. 2012). Mammals are the final hosts of liver fluke and are infected by consuming raw or uncooked freshwater fish harboring infective metacercariae. The developed adult worms live in the intrahepatic bile duct and cause pathological changes, such as hyperplasia and inflammation of biliary mucosa, periductal fibrosis, and even cholangiocarcinoma (CCA) development (Shin et al. 2010; Hong and Fang 2012; Sithithaworn et al. 2014).

The possible mechanisms of liver fluke-associated carcinogenesis include chronic irritation, nitric oxide (NO) formation, intrinsic nitrosation, and so on (Watanapa and Watanapa 2002). Our recent study has found a strong inducible nitric oxide synthase (iNOS) expression in the hepatobiliary tissues of mice infected with *C. sinensis* (in publishing data). The iNOS is originally expressed in the liver and inducible by a combination of lipopolysaccharide (LPS) and certain cytokines (Geller et al. 1993). INOS is responsible for the production of large quantities of NO upon stimulation under pathologic conditions (Green et al. 1994; Ahvazi et al. 1995). However, no report has been issued about the dynamics and mechanisms of iNOS/NO responses induced by *C. sinensis* infection.

Spleen is an important peripheral lymphoid organ in mammals and is the main filter for blood-borne pathogens and antigens. Spleen has a fundamental role in regulation of immune responses locally and in the whole body (Tarantino et al. 2013; Bronte and Pittet 2013). Studies have shown that spleen is an important place for iNOS expression in endotoxic shock (Kan et al. 2004). INOS is induced by inflammatory mediators in IRF1 and nuclear factor kappa-light-chain-enhancer of activated B cells (NF-κB)-dependent manner and responsible for high and prolonged NO production (Green et al. 1994; Kuhr et al. 2010). NO has demonstrated controversial roles both in immunosuppression during parasitic infections (Ahvazi et al. 1995) and control of blood parasitemia and parasitism (Vieira et al. 2009). Highly concentrated NO participates in the formation of peroxynitrite to nitrite (ONO_2_^−^), which can induce cytogenetic toxic effects and may correlate with the process of tumorigenesis (Szabó et al. 2007).

Previous studies have proven that BALB/c mice are susceptible to *C. sinensis* infection (Choi et al. 2003; Fu et al. 2008). In this experiment, we will study the iNOS and associated cytokine expression patterns in the spleen of mice with different infection intensities of *C. sinensis*. In addition, we will assess the role of spleen in peroxynitrite and nitrite responses, as well as the possible mechanisms of immunosuppression and development of CCA corresponding to iNOS/NO responses during infection.

## Materials and methods

### Metacercariae isolation

The metacercariae of *C. sinensis* were collected from *Pseudorasbora parva* captured in the Wang Tian Tang reservoir, Heng County, an endemic *C. sinensis*-infected area in Guangxi, China. The whole flesh of the fish was digested with artificial gastric juice, and the metacercariae of *C. sinensis* were collected as described previously (Yang et al. 2014). The metacercariae of *C. sinensis* were kept in 0.9 % NaCl and 4 °C until inoculation.

### Animals and infection

Specific pathogen-free (SPF) BALB/c mice (female) at 5 weeks of age were obtained from Guangxi Laboratory Animal Centre (GLAC, China) and further bred in the barrier facility at this center. Groups of 24 to 30 animals were inoculated with 20, 40, or 80 *C. sinensis* metacercariae in 100 μL of 0.9 % NaCl by oral gavage. Mice gavaging with 0.9 % NaCl were used as uninfected controls. All mice were fed sterile food and water during the experiment. The animals were checked daily until sample collection at 10, 20, 45, and 90 days post-infection (dpi). All animal study protocols were approved by the Institutional Animal Care and Use Committees of Guangxi.

### Plasma collection and detection of IgG antibodies against *C. sinensis*

Blood samples were collected from murine orbital sinus using a pipette into BD vacutainer tubes (3.6 mg K_2_ EDTA per tube). The plasma was separated by centrifugation and stored at −80 °C. An enzyme-linked immunosorbent assay (ELISA) kit (Combined Biotech. Co., Ltd., Shenzhen, China) with additional HRP-conjugated goat anti-mouse IgG antibody (Sangon, Shanghai, China) was used to detect the IgG antibodies against *C. sinensis* in the plasma. Briefly, murine plasma was diluted to 1:100 in phosphate-buffered saline (PBS) (pH 7.4) containing 5 % BSA and added into the antigen-coated plates (combined). After incubating at 37 °C for 30 min, the plates were washed four times with PBS (pH 7.4) containing 0.05 % Tween 20 (PBST) and then incubated with HRP-conjugated goat anti-mouse IgG antibody at 1:1000 dilution and 37 °C for 30 min. Bound secondary antibodies were allowed to react with H_2_O_2_-TMB substrate solutions (combined) at 37 °C for 10 min in the dark. Afterward, the reactions were stopped with 1 M H_2_SO_4_ and analyzed spectrometrically at 450 nm in detection and 630 nm in reference (Multiskan MK3, Thermo Fisher Scientific) to obtain the optical density (OD) value.

### Immunohistochemistry assay

Half of each spleen was obtained and fixed with 4 % formaldehyde-PBS (pH 7.4) for immunohistochemical analyses of iNOS expression. The paraffin-embedded tissues were cut into 4-μm-thick sections and placed on adhesion microscope slides (CITOGLAS, Haimen, China). The sections were baked at 60 °C for 30 min and then sequenced by soaking with dimethylbenzene (three times for 5 min), absolute alcohol (twice for 5 min), 95 % alcohol (twice for 5 min), 70 % alcohol (once for 5 min), and pure water (twice for 5 min) for dewaxing and dehydration. The sections were then treated with antigen retrieval reagents (Beyotime Institute Biotech, Haimen, China) by steaming for 20 min. After washing in immunol staining wash buffer (ISWB) (Beyotime) three times for 5 min, the sections were incubated with 3 % H_2_O_2_-methyl alcohol to inactivate endogenous peroxidase. After washing, the sections were incubated with immunol staining blocking buffer (ISBB) (Beyotime) at room temperature for 1 h. After removing the liquid, the sections were incubated with 1:100 diluted rabbit polyclonal antibody to iNOS (Abcam, Hong Kong) at 4 °C overnight. After washing, the sections were incubated with 1:50 diluted HRP-conjugated goat anti-rabbit IgG (H + L) (Beyotime) at room temperature for 1 h. After washing, the slides were stained with AEC horseradish peroxidase color development kit (Biotech Well, Shanghai, China) at room temperature for 15 min. After washing in pure water, the slides were air-dried and counterstained with hematoxylin solution (Sangon) at room temperature for 5 min and then washed again with pure water to stop the reaction. Finally, the slides were soaked in PBS (pH 7.4) to make blue color appear.

### Splenocyte isolation and total RNA extraction

Splenocytes were isolated by mouse lymphocyte separation medium (MLSM) (Dakewe Biotech Co., Ltd., Beijing, China) (density = 1.0810/mL, at 20 °C). Briefly, the spleens of the mice were washed carefully with PBS (pH 7.4) to remove the blood. Half of the spleen was then obtained and gently ground with RPMI 1640 (Invitrogen) using a syringe plunger to release the single splenocytes. The splenocytes were gently mixed and layered over 2 mL of MLSM and separated by centrifugation at 800×*g* for 15 min at room temperature. Splenocytes were collected from the MLSM-RPMI 1640 interface and then washed with PBS (pH 7.4) twice. The splenocyte pellets were resuspended in 200 μL of sample protector for RNA/DNA (TaKaRa, Dalian, China) and stored at −80 °C.

Total RNA samples were extracted from splenocytes through a single step using RNAiso Plus Reagent (TaKaRa). The RNA was isolated from the aqueous phase of the homogenized samples according to the protocol of the manufacturer; the RNA pellets were dissolved in 20 μL of RNase-free H_2_O.

### Real-time quantitative reverse transcriptase PCR

For real-time quantitative reverse transcriptase (qRT)-PCR, complementary DNA (cDNA) was synthesized by reverse transcription of total RNA, and genomic DNA was eliminated using PrimeScript™ RT Reagent Kit with gDNA Eraser (TaKaRa) according to the instructions of the manufacturer. Specific primers used in real-time assays were designed using the Primer-BLAST (http://www.ncbi.nlm.nih.gov/tools/primer-blast/index.cgi?LINK_LOC=BlastHome) and synthesized by Sangon Biotech Co., Ltd. (Shanghai, China) (Table 1). Previously published primers specific for mice β-actin (Gerard et al. 2002) were used to normalize the messenger RNA (mRNA) expression levels.

**Table 1:** Primers used in qRT-PCR assay

Specificity	Accession number	Primers	Sequences	Product size (bp)	Product Tm (°C)
β-Actin^a^	NM_007393	F	5′-GGCCGGGACCTGACAGACTACCTC-3′	90	84.8
		R	5′-GTCACGCACGATTTCCCTCTCAGC-3′		
iNOS	NM_010927	F	5′-TCTTGGAGCGAGTTGTGGATTGTC-3′	132	86.0
		R	5′-AGTAGGTGAGGGCTTGGCTGAGTG-3′		
IFN-γ	NM_008337	F	5′-AGGAACTGGCAAAAGGATGGTGAC-3′	109	80.6
		R	5′-TGTTGTTGCTGATGGCCTGATTGT-3′		
IL-12p35	BC116855	F	5′-AACATTATTCCTGCACTGCT-3′	86	80.9
		R	5′-CAGTGGTAAACAGGTCTTCA-3′		
IL-12p40	NM_008352	F	5′-GTAAGTTCTCTCCTCTTCCC-3′	130	85.1
		R	5′-GATGGTTAGCTTCTGAGGAC-3′		
IL-18	NM_008360	F	5′-ACCAAGTTCTCTTCGTTGAC-3′	94	80.3
		R	5′-ATTATCAGTCTGGTCTGGGG-3′		

^a^Reference to Gerard et al. 2002

Real-time PCRs were performed using SYBR® Premix Ex Taq™ kits (TaKaRa). Briefly, fixed Ex Taq DNA polymerase for “hot start,” DNA intercalated dye SYBR Green I, dNTP mixture, Mg^2+^ and 0.20 μM of gene-specific primers, 1 μL of cDNA and 0.4 μL of 50× ROX reference dye I were combined in a final volume of 20 μL. Amplification and data acquisition were carried out using an Eco™ Real-Time PCR system with Eco™ Software v3.0.16.0. (Illumina), with the following cycling parameters: predenaturation at 95 °C for 30 s, 45 cycles of amplification at 95 °C for 5 s, and annealing and extension at 60 °C for 20 s. This process was followed by melt curve analysis from 65 to 95 °C to display the melting temperature (Tm) (Table 1) for each of the identified specific DNA product populations. For quantification of each target gene, a novel relative quantitative method of real-time RT-PCR assay based on the slope of standard curve (Zhang et al. 2005) was used. Serially diluted cDNA samples were used as standards to prepare the standard curves and obtain the slopes. The relative levels of the target gene expression were calculated with reference to β-actin.

### Statistical analyses

Data are presented as means ± SEM. Paired-sample *t* tests using SPSS 11.5 for windows were used to test for significant differences in cytokine expression between groups. Comparisons were considered significant at *P* ≤ 0.05 and highly significant at *P* ≤ 0.01 or *P* ≤ 0.001.

## Results

### IgG antibody production in mice after *C. sinensis* infection

**Fig. 1:**
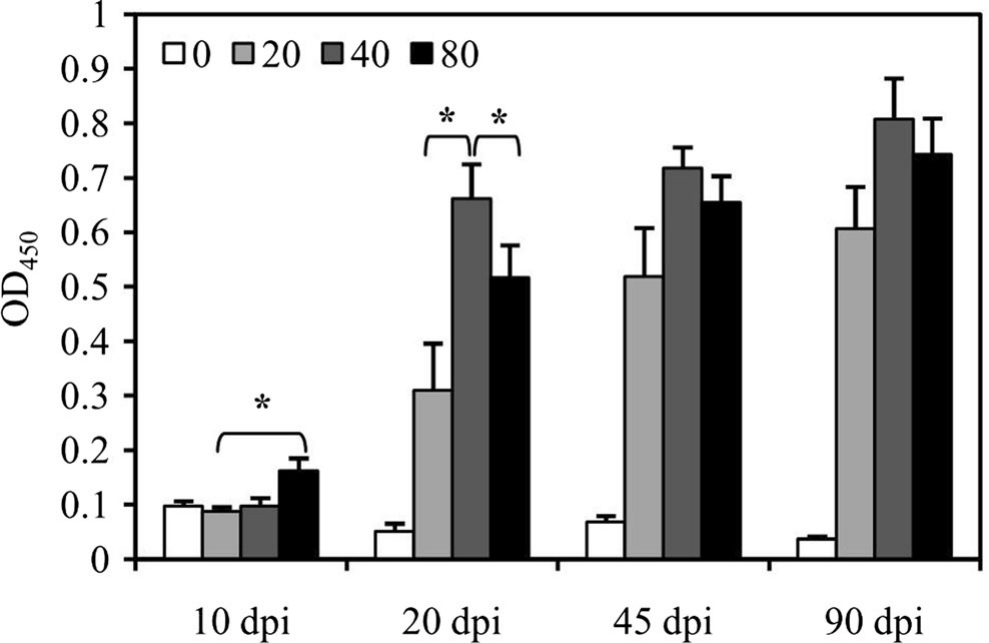
Specific IgG against *C. sinensis* levels in the plasma of mice. The mice were infected with 20, 40, or 80 *C. sinensis* metacercariae by gavage; uninfected mice were used as controls. The IgG antibodies against *C. sinensis* were detected by ELISA at 10, 20, 45, and 90 dpi. The relative levels of the specific IgG antibodies were expressed with OD_450_ value. *P < 0.05. *dpi* days post-infection

The levels of IgG antibodies against *C. sinensis* slightly increased in the plasma of the mice inoculated with 80 metacercariae as early as 10 dpi and significantly increased after 20 dpi, reaching the highest levels at 90 dpi in this experiment. Mice gavaging with 20 or 40 metacercariae also showed increased levels of IgG antibodies against *C. sinensis* after 20 dpi. Higher levels of IgG antibodies were induced by inoculating with 40 metacercariae than that with 20 or 80 metacercariae during infection, especially at 20 dpi (*P* < 0.05) (Fig. 1).

### INOS expression in the spleen of mice after *C. sinensis* infection

**Fig. 2:**
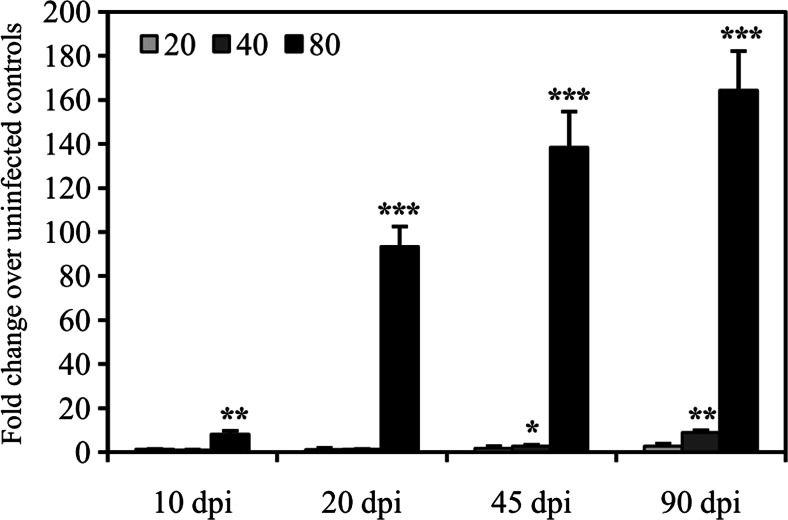
INOS mRNA transcription in the splenocytes of mice infected with *C. sinensis*. The mice were infected with 20, 40, or 80 *C. sinensis* metacercariae by gavage. The transcript levels were detected at 10, 20, 45, and 90 dpi using qRT-PCR and expressed as fold change over uninfected controls with SEM bars. Significance was determined by comparison with the uninfected control groups: *P < 0.05, **P < 0.01, ***P < 0.001. *dpi* days post-infection

The relative levels of iNOS mRNA transcription in the splenocytes of mice infected with *C. sinensis* were detected using qRT-PCR at 10, 20, 45, and 90 dpi. The iNOS transcripts gradually increased by 8.13- (*P* < 0.01), 93.4- (*P* < 0.001), 138.5- (*P* < 0.001), and 164.4-fold (*P* < 0.001) above the uninfected controls when infected with 80 metacercariae at 10, 20, 45, and 90 dpi, respectively. The transcripts of iNOS also increased in mice infected with 40 metacercariae at 45 and 90 dpi by 2.74- (*P* < 0.05) and 8.81-fold (*P* < 0.01), whereas no changes in iNOS mRNA transcription were detected in the splenocytes of mice infected with 20 metacercariae in this study (Fig. 2).

**Fig. 3:**
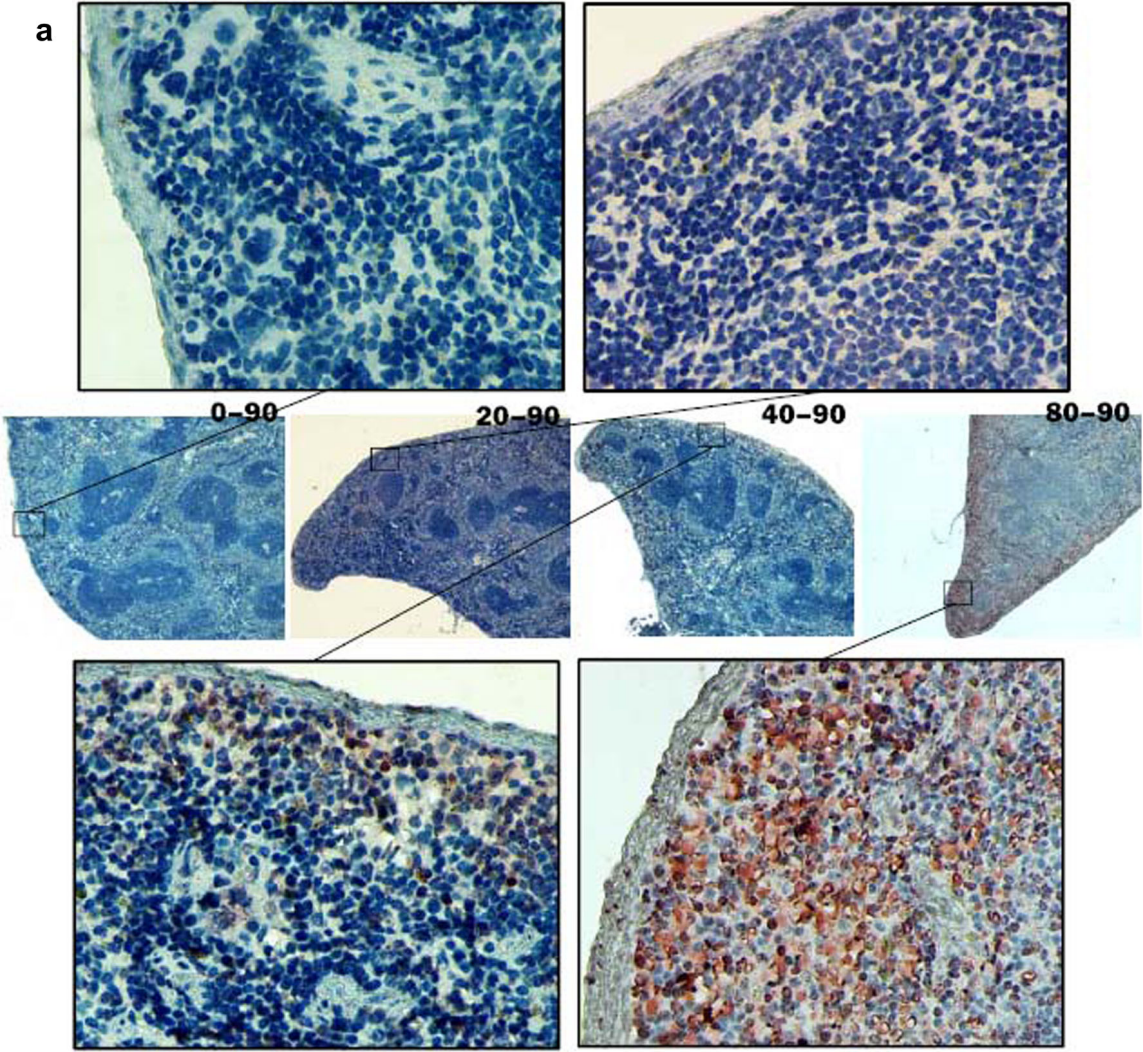

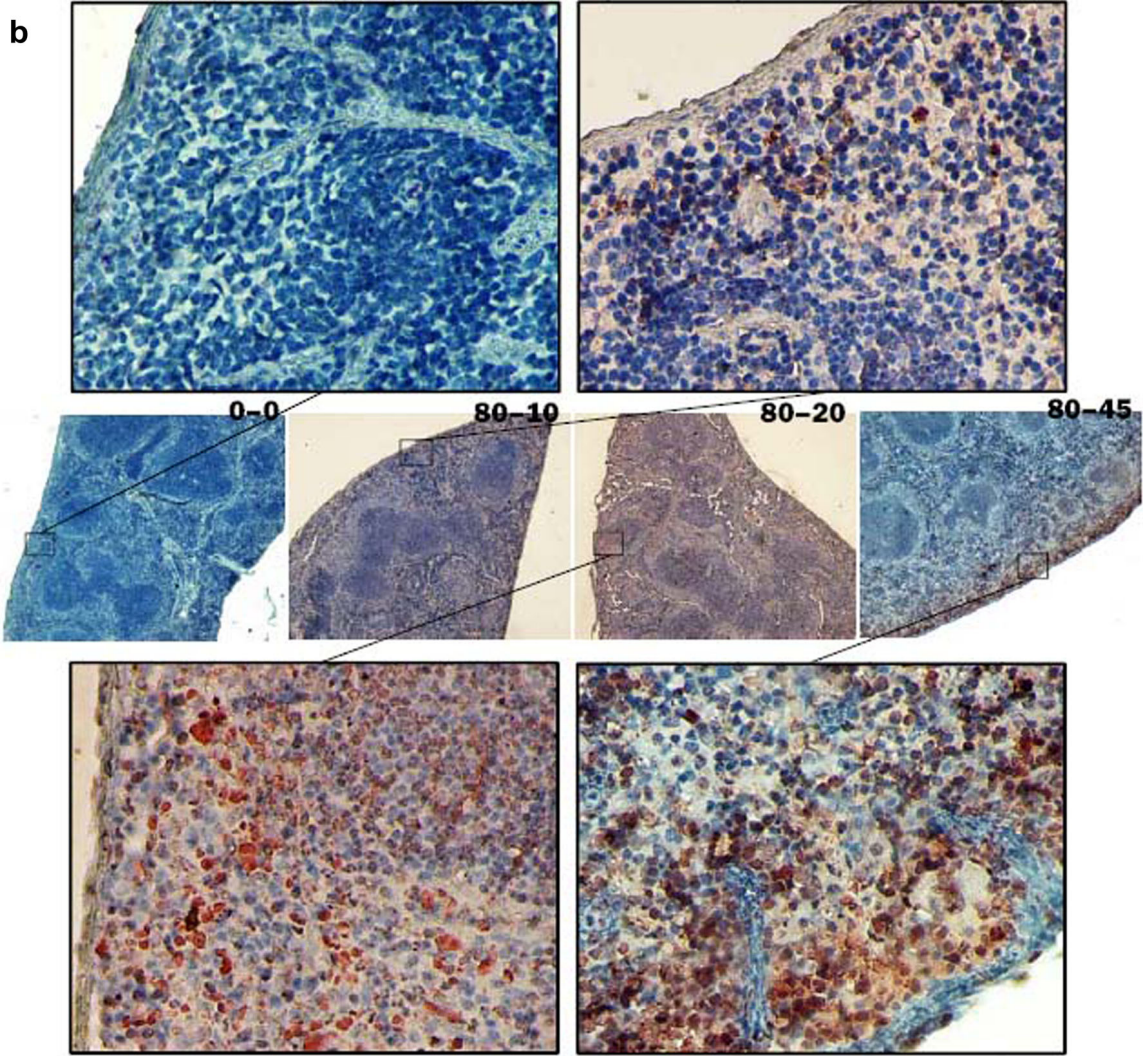
INOS expression in the spleen of mice infected with *C. sinensis*. Slices of the spleens were incubated with iNOS antibody and HRP-conjugated goat anti-rabbit IgG (H + L) by sequence and then processed with AEC color development reagent to show the iNOS expression in the cytoplasm of splenocytes with *red color*. Finally, the slices were counterstained with hematoxylin to display the splenocyte nucleus with *blue*. **a** At 90 dpi, iNOS expressions were detected in the mice infected with 20, 40, or 80 metacercariae and the uninfected controls. The pictures were labeled as 20–90, 40–90, 80–90, and 0–90 accordingly. **b** Mice were infected with 80 metacercariae, and iNOS expression was detected at 10, 20, and 45 dpi. The uninfected mice were as controls. The pictures were labeled as 80–10, 80–20, 80–45, and 0–0 accordingly

The iNOS expression in the spleen of mice was also detected by immunohistochemical assay. Massive iNOS-expressed cells were detected in the spleen of mice infected with 80 metacercariae at 90 dpi. A slight iNOS expression was detected in the spleen of mice infected with 40 metacercariae, whereas no remarkable iNOS expression was detected in normal mice or those infected with 20 metacercariae at 90 dpi (Fig. 3a). In the mice infected with 80 metacercariae, the iNOS-expressed splenocytes appeared as early as 10 dpi and then remarkably increased at 20 and 45 dpi (Fig. 3b). The iNOS-expressed cells were mainly located in the red pulp and appeared more intensive near the capsule (Fig. 3a, b).

### Effect of *C. sinensis* infection on IFN-γ, IL-12, and IL-18 mRNA transcription in splenocytes

**Fig. 4:**
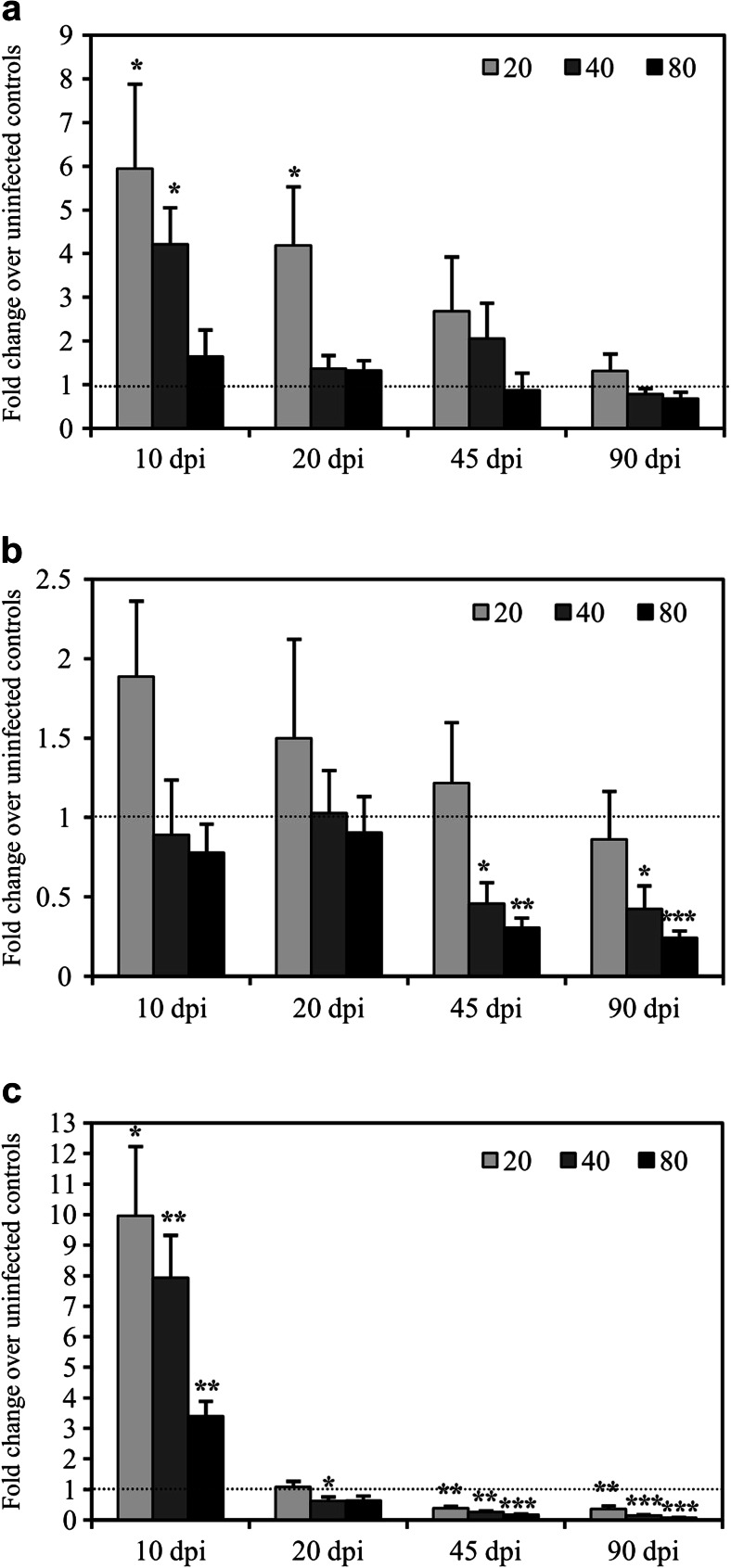
Quantification of IFN-γ, IL-12p35, and IL-18 transcripts in the splenocytes of mice infected with *C. sinensis*. BALB/c mice were infected with 20, 40, or 80 *C. sinensis* metacercariae by gavage. The levels of IFN-γ (**a**), IL-12p35 (**b**), and IL-18 transcript (**c**), were detected at 10, 20, 45, and 90 dpi and expressed as fold change over uninfected controls with SEM bars. The *dotted lines* represent 1-fold change (or no change) over the normal controls. Significance was determined by comparison with uninfected control groups: *P < 0.05, **P < 0.01, ***P < 0.001. *dpi* days post-infection

Splenocytes from the mice infected with *C. sinensis* were detected in vitro using qRT-PCR for interferon (IFN)-γ, IL-12, and IL-18 mRNA expressions at 10, 20, 45, and 90 dpi. IFN-γ mRNA transcription substantially increased by 4.21- (*P* < 0.05) and 4.19-fold (*P* < 0.05) above the uninfected control background in mice infected with 20 metacercariae at 20 and 45 dpi but decreased to normal levels at 90 dpi. The IFN-γ transcript level increased by 5.94-fold (*P* < 0.05) in mice infected with 40 metacercariae at 20 dpi but subsequently decreased to normal levels after 45 dpi. No differences for IFN-γ transcripts were observed both in the infected groups at 10 dpi and in the mice inoculated with 80 metacercariae during the experiment (Fig. 4a).

No differences were observed for IL-12p35 mRNA transcription in splenocytes of mice inoculated with different amounts of metacercariae at 10 and 20 dpi. However, significant decreases by 0.31- (*P* < 0.01) and 0.46-fold (*P* < 0.05) were observed in mice infected with 40 and 80 metacercariae at 45 dpi, respectively, and 0.43- (*P* < 0.05) and 0.24-fold (*P* < 0.001) at 90 dpi, respectively. Similarly, no changes were observed for IL-12p35 mRNA transcription in the splenocytes of mice infected with 20 metacercariae at 45 and 90 dpi (Fig. 4b). Unlike the background expression of IL-12p35 mRNA, very low backgrounds of IL-12p40 mRNA expression were observed in the uninfected controls. The significant decreases of IL-12p40 mRNA expression were manifested to an undetected level by qRT-PCR after *C. sinensis* infection in this experiment (data not shown).

Meanwhile, IL-18 mRNA transcription increased by 3.40- (*P* < 0.01), 9.96- (*P* < 0.05), and 7.93-fold (*P* < 0.01) in the splenocytes of mice infected with 20, 40, and 80 metacercaria at 10 dpi, respectively, and significantly decreased after 20 dpi. IL-18 transcripts were significantly reduced by 0.15- (*P* < 0.001), 0.08- (*P* < 0.001), and 0.36-fold (*P* < 0.01) at 20 dpi, respectively, and then by 0.18- (*P* < 0.001), 0.38- (*P* < 0.01), and 0.26-fold (*P* < 0.01) at 45 dpi, respectively, and finally, by 0.64-, 0.62- (*P* < 0.05), and 1.08-fold at 90 dpi, respectively, in this experiment (Fig. 4c).

## Discussion

The pathological responses occurring in bile duct tissues have been identified as the characteristic lesions during *C. sinensis* infection (Choi et al. 2003; Fu et al. 2008; Hong and Fang 2012). Our resent study has observed a specific pathological response and iNOS expression pattern in hepatobiliary tissues after *C. sinensis* infection (in-publishing data).

The spleen is a site where innate and adaptive immune responses against pathogens are initially induced and regulated. Early studies have found that the splenic macrophages from *Histoplasma capsulatum*-infected mice express iNOS, which correlates with the severity of the infection (Wu-Hsieh et al. 1998). Higher levels of iNOS mRNA transcription in the spleen of C57BL/6 mice have been observed during early infection with blood-stage *Plasmodium chabaudi*, which correlates with resistance against the parasite (Jacobs et al. 1995). Studies have also demonstrated that dogs infected with *Trypanosoma cruzi* or suffered visceral leishmaniasis exhibit increased iNOS expression in the spleen and lymph node, which is associated with clinical worsening of the disease and high parasitism (Vieira et al. 2009; dos Santos et al. 2011; Sanches et al. 2014). In this study, we found that spleen is also an important organ for iNOS/NO production during *C. sinensis* infection.

The BALB/c mice have been confirmed to be susceptible to *C. sinensis* infection but are no match for the FVB mice (Choi et al. 2003; Fu et al. 2008). In this study, we have also confirmed that BALB/c mice are sensitive to liver fluke infection and exhibit significant increase in specific IgG antibodies against *C. sinensis*. Compared with FVB mice, BALB/c mice exhibit relatively higher level of IFN-γ and lower level of IL-4 production in spleen cells after *C. sinensis* infection (Choi et al. 2003). In this study, we have found that the BALB/c mice exhibit increased IFN-γ transcript in splenocytes when challenged with mild or moderate amount of *C. sinensis* metacercariae at the early stage of infection. However, this increase in IFN-γ transcript decreased to almost normal level after 45 dpi, which is in accordance with the result that IFN-γ production decreased between 2 and 4 weeks in both BALB/c and FVB mice infected with *C. sinensis* (Choi et al. 2003). However, no positive correlation has been observed between IFN-γ transcription and iNOS expression in splenocytes during *C. sinensis* infection in the experiment. Surprisingly, the mice suffering severe infection did not exhibit an increase in the IFN-γ transcription but showed strong iNOS expression in the splenocytes. Therefore, we considered that iNOS expression in the splenocytes could not attribute to the dominance of Th1-type cytokines after *C. sinensis* infection.

The iNOS is originally described as an enzyme induced by endotoxins and a wide group of cytokines and mainly expressed in activated macrophages and microglia (Jablonska et al. 2008; Nahrevanian 2009; Yang et al. 2011). Studies have demonstrated that higher amounts of NO and iNOS gene expression in splenocytes can be induced by cyclophosphamide (CTX) and estrogen treatment and mediated in part by IFN-γ (Angulo et al. 2000; Karpuzoglu et al. 2006). The IFN-γ, coupled with IL-12 and IL-18, has been considered as the key factor for inducing iNOS/NO production both in vivo and in vitro (Schindler et al. 2001; Dinarello 2002; Kawakami et al. 2004; Dinarello et al. 2013). The synthesis of IFN-γ is critically regulated by IL-12, IL-18, and IL-23 (Kawakami et al. 2004). IL-12 can promote the Th1 pathway by stimulating the production of IFN-γ by NK cells and T cells (Jakobi and Petry 2008). IL-18 was considered as a primary proinflammatory cytokine because of its ability to stimulate the expression of genes associated with inflammation. IL-18 could also induce IFN-γ, in combination with IL-12 or IL-15, and has been proved to be an “IFN-γ-inducing factor” (Mullen et al. 2006; Dinarello 2002; Dinarello et al. 2013).

The regulation networks among IFN-γ, IL-12, IL-18, and iNOS/NO in innate immunity are complex. IFN-γ production by various cell types requires different signals. IL-12 alone induces the expression of IFN-γ mRNA; however, the release of IFN-γ protein and the subsequent production of NO are strictly dependent on the simultaneous presence of IL-18 (Schindler et al. 2001). IFN-γ treatment increases the levels of IL-12 and iNOS/NO production, which is paralleled by a concomitant increase in antileishmanial activity (Das et al. 2001). Meanwhile, IL-12 shows an IFN-γ-dependent, iNOS-inducing activity and promotes tumor regression through activation of multiple lymphocytic and myelocytic effectors (Egilmez et al. 2011). The function of NO for IL-12 expression is also complex. Early studies have suggested that NO can induce transcription of IL-12p40, but not IL-12p35, in human macrophages (Salvucci et al. 1998). The IL-12p40 homodimer is an antagonist for IL-12, and this antagonism may be at least partially responsible for reduced Th1 reactivity in the presence of NO (Pahan et al. 2001). However, a latter report has indicated that the IL-12p40 homodimer can also induce NO production by microglia (Jana et al. 2009). Our recent study has also demonstrated a negative correlation between iNOS/NO responses and an unbalanced IL-12p40/IL-12p35 transcription pattern, the very high level of IL-12p40 accompanied by the very low level of IL-12p35 transcript in microglia infected with Marek’s disease virus (Yang et al. 2011).

Meanwhile, highly concentrated iNOS produced by antigen-presenting cells (APCs) may inhibit IL-12 synthesis, thereby contributing to desensitization of macrophages after exposure to inflammatory stimuli (van der Veen 2001). Furthermore, NO affects the immune profile of Th1 cells because mice with a disrupted iNOS gene exhibit enhanced Th1 activity (Singh et al. 2000). Thus, we considered that the highly expressed iNOS in the splenocytes may regulate Th1 response negatively and affect the Th1/Th2 balance after *C. sinensis* infection. Studies have demonstrated that many worms, including *Trichinella spiralis* (Wandurska-Nowak and Wiśniewska 2002), *Toxocara canis* (Espinoza et al. 2002), *Echinococcus granulosus* (Amri et al. 2007), and *Paragonimus westermani* (Jin et al. 2009), significantly induce iNOS/NO responses in immunocompetent cells, and these inductions may constitute both defense and evasion/adaptation mechanisms of the parasites.

The pathogen-associated molecular patterns (PAMPs) recognized by pattern recognition receptors (PRRs) activate certain intracellular signaling pathways and promote proinflammatory cytokines and iNOS expression (Yang and Wei 2008; Yang and Shen 2013). Although PAMPs from *C. sinensis* have not been identified, clues of PAMPs existing in the excretory-secretory products (ESPs) of *C. sinensis* have been reported (Pak et al. 2009). ESPs induce the expression of proinflammatory cytokines, IL-1β and IL-6, and the iNOS/NO responses in an NF-κB-dependent manner (Nam et al. 2012). A recent study has found that *C. sinensis* ferritin heavy chain (*Cs*FHC), which has been previously confirmed as a component of ESPs, exhibits stimulatory properties in activation of NADPH oxidase, xanthine oxidase, and iNOS (Mao et al. 2014).

In addition, IL-12-induced iNOS/NO responses in macrophages have been considered a novel mechanism of macrophage suppression (Yim et al. 2013), which is primarily mediated by IL-10 in certain parasite infection (Sadler et al. 2003). Studies have demonstrated that immunosuppression is a prominent characteristic of the host-parasite interplay. The spleen cells and intraperitoneal macrophages from *Echinococcus multilocularis*-infected mice express significantly increased levels of iNOS, which results in NO production and mediates immunosuppression (Dai and Gottstein 1999; Dai et al. 2003). The fluke and the human body have adapted well to each other during *C. sinensis* infection, and most of the infected humans feel minimal subjective symptoms (Hong and Fang 2012). This permanent iNOS expression induced by *C. sinensis* may be responsible for immunosuppression and development of CCA.

These data have demonstrated that spleen is a crucial organ for iNOS expression, which may contribute a permanent and high level of NO production and subsequent peroxynitrite to nitrite reactions during *C. sinensis* infection. This iNOS expression does not depend on Th1 responses and may influence pathological responses in the hepatobiliary tissues of *C. sinensis*-infected hosts. These results have also provided a clue for the existence of PAMPs in *C. sinensis* and have led investigators to focus the roles of iNOS/NO and the associated mechanisms in both immunosuppression and development of CCA during clonorchiasis.

## Abbreviations

APCs: Antigen-presenting cells
CCA: Cholangiocarcinoma
CTX: Cyclophosphamide
dpi: Days post-infection
ESPs: Excretory-secretory products
IHC: Immunohistochemistry
iNOS: Inducible nitric oxide synthase
IL: Interleukin
ISBB: Immunol staining blocking buffer
ISWB: Immunol staining wash buffer
LPS: Lipopolysaccharide
MLSM: Mouse lymphocyte separation medium
NO: Nitric oxide
PAMPs: Pathogen-associated molecular patterns
PBS: Phosphate-buffered saline
PRRs: Pattern recognition receptors
SPF: Specific pathogen free
TLR: Toll-like receptors
